# Nodular vulvar lesions and its key differential diagnoses: a case report

**DOI:** 10.31744/einstein_journal/2025RC0362

**Published:** 2025-03-10

**Authors:** Juliana Jorge Romano, Fernanda Kesselring Tso, Yasmin Cristina Cesquim Noronha, Julizia Foloni May, Mariano Tamura Vieira Gomes, Sérgio Podgaec, Patricia Napoli Belfort-Mattos

**Affiliations:** 1 Hospital Israelita Albert Einstein São Paulo SP Brazil Hospital Israelita Albert Einstein, São Paulo, SP, Brazil.

**Keywords:** Vulvar neoplasms, Adenoma, sweat gland, Sweat gland neoplasms, Diagnosis, differential, Gynecological examination

## Abstract

The etiologies of vulvar nodules range from infectious to neoplastic. Owing to the wide spectrum of vulvar diseases, clinical differentiation of the lesions is crucial for adequate diagnosis and management. A 46-year-old patient presented with a nodular lesion in the vulvar region that had been growing slowly for 10 years. Gynecological examination revealed the presence of a solid nodular lesion measuring approximately 3 cm in the middle third of the right labia majora without phlogistic signs or secretion discharge. The lesion was excised and an anatomopathological study revealed a nodular hidradenoma. Nodular hidradenoma is a rare benign neoplasm of the apocrine glands that mainly affects women between 30 and 60 years of age. Although generally asymptomatic, some symptoms have been described, such as itching, pain, ulceration, or secretory discharge. The diagnosis was made by an anatomopathological study, and treatment consisted of total excision of the lesion without the need to enlarge the margin. During the investigation, malignant lesions such as liposarcoma of the vulvar region and non-melanoma skin cancers were excluded. Differential diagnosis is important because it requires a more invasive approach and surgical margins. This report aimed to expand the knowledge of the diagnosis of vulvar hidradenoma to exclude the possibility of malignant neoplasia while caring for vulvar nodules, thereby allowing proper management of the case.

## INTRODUCTION

Vulvar nodules present with a diverse spectrum of etiologies that range from infectious conditions to various neoplasms.^1-3^ These differential diagnoses span a broad array of possibilities including bacterial Bartholin’s gland abscess, pyoderma gangrenosum, abscess formation, papillary hidradenoma, verrucous lesion, sebaceous cyst, lipoma, neurofibroma, and even malignant tumors, such as metastatic papillary carcinoma, papillary syringocystadenocarcinoma, and squamous cell carcinoma.^4,5^

Management of vulvar nodules has garnered increasing attention driven by concerns regarding malignant neoplasms within the genital region. Within this context, and recognizing the imperative for comprehensive therapeutic solutions addressing both medical and aesthetic aspects, the importance of reporting and addressing such cases cannot be overstated.^6^

In this context, we present a case report that addresses the diagnosis and treatment of vulvar nodules while also highlighting the clinical implications of these conditions. By sharing this case, our aim was to contribute to the collective knowledge and promote effective clinical strategies for managing similar challenges in gynecology and dermatology.^3,6^ Thus, we provide a brief review of the main differential diagnoses for vulvar nodules.

## CASE REPORT

A 46-year-old woman was referred for evaluation to the Lower Tract Pathology outpatient clinic of *Hospital M’Boi Mirim* due to the presence of a nodular lesion in the middle third of the labia majora that had been progressively growing over a period of 10 years. She denied experiencing fever, bleeding, vaginal discharge, or local pruritus.

The patient had controlled chronic arterial hypertension and underwent a partial gastrectomy in February 2020 for gastric adenocarcinoma. She presented with an eumenorrheic menstrual cycle induced by medroxyprogesterone. Additionally, she reported no history of sexually transmitted infections (STIs). She had four pregnancies, including three previous cesarean sections and one miscarriage.

**Figure 1:**
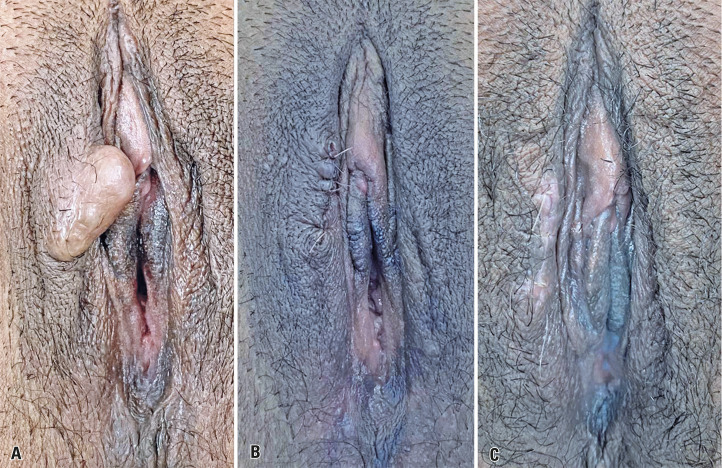
A) Nodular lesion of solid consistency of approximately 3 cm, located in the middle third of the right labia majora; B) Immediate post-op of vulvar lesion exeresis; C) 30 days after vulvar lesion excision surgery

Gynecological examination revealed a nodular lesion with a solid consistency measuring approximately 3 cm in diameter. The lesion was located in the middle third of the right labia majora, and showed no signs of inflammation or secretory discharge (Figure 1A).

**Figure 2:**
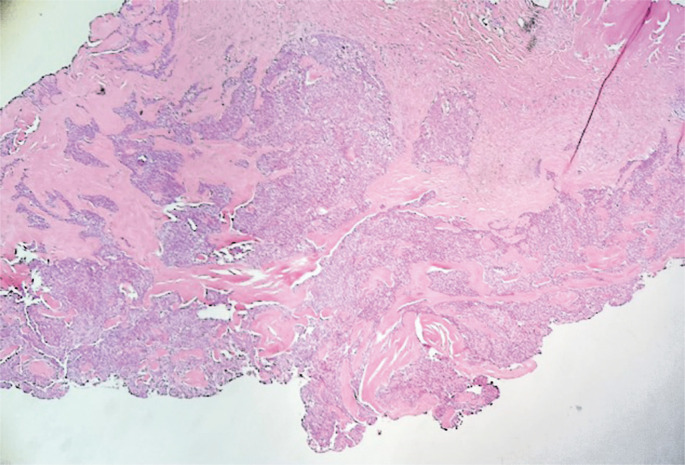
Histopathological analysis – well-circumscribed, dermal-based tumor with solid areas. Solid areas composed of varying proportion of clear cells, poroid cells, and squamoid cells. Ducts with eosinophilic cuticles present in solid areas. Stroma between solid islands is prominently hyalinized-appearing

In the operating room, the nodular lesion was excised under spinal anesthesia using a high-frequency scalpel equipped with a needle electrode (45 watts power-pure cut). An arcuate longitudinal incision was made to excise the lesion and capsule with adequate hemostasis. After opening the capsule, a moderate amount of solid, pasty, yellowish, and foul-smelling material was expelled. After excision, layered suturing was performed with edge approximation using PDS 4.0 sutures (Figure B). The patient had a good postoperative recovery without complications (Figure C). Anatomopathological examination confirmed the diagnosis of a nodular hidradenoma (Figure 2).

This study was approved by the Ethics Committee of the *Hospital Israelita Albert Einstein* (CAAE: 62042322.6.0000.0086; # 5.716.711). Informed consent was obtained from all patients included in this study.

## DISCUSSION

Nodular hidradenoma, also known as tubular hidradenoma, is a rare benign neoplasm originating from the apocrine glands that primarily affects women aged between 30 and 60 years.^1^ While its most common location is the vulva, it may also manifest in other areas such as the breast, armpits, inguinal, and perianal regions.^2^ It typically presents as a small, asymptomatic, and slow-growing nodule within the dermis or subcutaneous tissue of the female anogenital region. These nodules are typically singular, mobile, and well defined. Although generally asymptomatic, nonspecific symptoms including itching, pain, bleeding, ulcerations, and secretions can occur.^2,4^

Adenoma cells within nodular hidradenomas contain estrogen, progesterone, and testosterone receptors, thereby suggesting hormonal control of the lesions. The clinical presentation may be exacerbated during the menstrual period in premenopausal individuals, with symptom stabilization after menopause. This has led to occasional comparisons with ductal papillomas in breast tissue.^4^ Diagnosis relies on histological examination, which reveals a partially solid or solid cystic dermal lesion with papillary projections associated with columnar secretory cells, flattened myoepithelial cells, and dilated cystic spaces.^6^

Treatment consisted of complete excision of the lesion without the need for a margin. In cases where total excision was not achieved, the recurrence rate was 12%. Complete excision, recurrence, and progression to adenocarcinoma are rare. Associated comorbidities may occur despite the low incidence. There is one case described in the literature of a hidradenoma associated with extramammary Paget’s disease^7,8^ and another with concurrent vulvar melanoma.^9^ In both instances, the authors regarded the findings as accidental and lacked direct association with vulvar hidradenomas.

Among the differential diagnoses of nodular hidradenoma, the primary consideration is papillary syringocystadenoma, which is a benign adnexal neoplasm that frequently demonstrates apocrine differentiation.^5^ This condition typically presents as a solitary plaque or nodule, usually on the scalp and face, although it is occasionally found in atypical locations, such as the vulva. The recommended approach for both conditions is complete excision of the lesion. It is imperative to rule out other significant differential diagnoses, including malignant lesions such as vulvar liposarcoma. Liposarcomas in the vulvar region typically present as a single small- or medium-sized lesion. Their well-circumscribed macroscopic appearance often contributes to the misdiagnosis of benign conditions.^10^

Additionally, nonmelanoma skin cancers, including basal cell and squamous cell carcinomas, warrant further consideration. These cancers typically exhibit rapid growth, associated vascularization, and symptoms such as pruritus, secretion discharge, and ulceration. Recognizing this differential diagnosis is crucial, as it requires a more invasive approach with a surgical excision margin of 3-5 mm for basal cell carcinoma and 4-6 mm for squamous cell carcinoma.

## CONCLUSION

Accurate diagnosis of vulvar tumors is crucial to ensure appropriate treatment decisions, avoid invasive procedures for benign lesions, and prevent mismanagement of malignant lesions, which could lead to significant harm to the patient.
